# Extended-spectrum β-lactamases in Gram Negative Bacteria

**DOI:** 10.4103/0974-777X.68531

**Published:** 2010

**Authors:** Deepti Rawat, Deepthi Nair

**Affiliations:** *Department of Microbiology, Maulana Azad Medical College, New Delhi, India*; 1 *Department of Microbiology, Vardhaman Mahavir Medical College & Safdarjang Hospital, New Delhi, India*

**Keywords:** Extended-spectrum β-lactamases, Gram negative bacteria (GNB) and Antimicrobial resistance

## Abstract

Extended-spectrum β-lactamases (ESBLs) are a group of plasmid-mediated, diverse, complex and rapidly evolving enzymes that are posing a major therapeutic challenge today in the treatment of hospitalized and community-based patients. Infections due to ESBL producers range from uncomplicated urinary tract infections to life-threatening sepsis. Derived from the older TEM is derived from Temoniera, a patient from whom the strain was first isolated in Greece. β-lactamases, these enzymes share the ability to hydrolyze third-generation cephalosporins and aztreonam and yet are inhibited by clavulanic acid. In addition, ESBL-producing organisms exhibit co-resistance to many other classes of antibiotics, resulting in limitation of therapeutic option. Because of inoculum effect and substrate specificity, their detection is also a major challenge. At present, however, organizations such as the Clinical and Laboratory Standards Institute (formerly the National Committee for Clinical Laboratory Standards) provide guidelines for the detection of ESBLs in Klebsiella pneumoniae, *K. oxytoca, Escherichia coli* and *Proteus mirabilis*. In common to all ESBL-detection methods is the general principle that the activity of extended-spectrum cephalosporins against ESBL-producing organisms will be enhanced by the presence of clavulanic acid. Carbapenems are the treatment of choice for serious infections due to ESBL-producing organisms, yet carbapenem-resistant isolates have recently been reported. ESBLs represent an impressive example of the ability of gram-negative bacteria to develop new antibiotic-resistance mechanisms in the face of the introduction of new antimicrobial agents. Thus there is need for efficient infection-control practices for containment of outbreaks; and intervention strategies, e.g., antibiotic rotation to reduce further selection and spread of these increasingly resistant pathogens.

## INTRODUCTION

Extended-spectrum β-lactamases (ESBLs) are a rapidly evolving group of β-lactamases which share the ability to hydrolyze third-generation cephalosporins and aztreonam but are inhibited by clavulanic acid. They represent the first example in which β-lactamase–mediated resistance to β-lactam antibiotics resulted from fundamental changes in the substrate spectra of the enzymes.[1]

The total number of ESBLs now characterized exceeds 200. These are detailed on the authoritative website on the nomenclature of ESBLs hosted by George Jacoby and Karen Bush (http://www.lahey.org/studies/webt.htm). Published research on ESBLs has now originated from more than 30 different countries, reflecting the truly worldwide distribution of ESBL-producing organisms.

Enterobacteriaceae, especially *Klebsiella spp*.-producing ESBLs such as SHV and TEM types, have been established since the 1980s as a major cause of hospital-acquired infections. However, during the late 1990s, several community-acquired pathogens that commonly cause urinary tract infections and diarrhea have also been found to be ESBL producers. These include *Escherichia coli, Salmonella, Shigella and Vibrio cholerae*.[2–4]

ESBLs are often encoded by genes located on large plasmids, and these also carry genes for resistance to other antimicrobial agents such as aminoglycosides, trimethoprim, sulphonamides, tetracyclines and chloramphenicol.[5] Recent studies have demonstrated fluoroquinolone resistance mediated by co-transfer of the *qnr* determinant on ESBL-producing plasmids.[6,7] Thus, very broad antibiotic resistance extending to multiple antibiotic classes is now a frequent characteristic of ESBL-producing enterobacterial isolates. As a result, ESBL-producing organisms pose a major problem for clinical therapeutics. This review attempts to present a comprehensive picture on the basis of the currently available literature about this diverse, complex and rapidly evolving group of enzymes.

## RESISTANCE TO β-LACTAMS

β-Lactams are a group of antibiotics acting on the cell wall of a bacterial cell. These include the penicillins, cephalosporins, carbapenems and monobactems. These bind to and inhibit the carboxypeptidases and transpeptidases. These are the cell wall synthesizing enzymes, also called the penicillin-binding proteins, or PBPs, that catalyze the D-ala D-ala cross linkages of the peptidoglycan wall that surrounds the bacterium. As a result, there is weakening of the cell wall structure, leading to cell lysis.

Resistance to β-lactams has probably arisen throughout bacterial history but has become a useful and therefore selected trait since the β-lactam antibiotics came into clinical use. These drugs exerted a Darwinian selection, killing susceptible bacteria and allowing the resistant ones to survive.

Resistance to β-lactams may be inherent to a particular species, as seen in enterococci, which have inherently insensitive PBPs. Alternately, it may be acquired through spontaneous mutation or DNA transfer. Functionally, β-lactam resistance may be a result of the production of β-lactamases, impermeability, efflux and target modification. These modalities may occur singly or in different combinations.

The most common causes of resistance in gram-positive cocci like pneumococci and MRSA are changes in the normal PBPs or acquisition of additional β-lactam–insensitive PBPs. However, in the gram-negative bacteria, resistance is mostly due to a combination of endogenous acquired β-lactamases, along with natural up-regulated impermeability and efflux.[8]

## DEFINITION OF EXTENDED-SPECTRUM β-LACTAMASES

There is no consensus on the precise definition of ESBLs. A commonly used working definition is that, ESBLs are β-lactamases capable of conferring bacterial resistance to the penicillins; first-, second- and third-generation cephalosporins; and aztreonam (but not the cephamycins or carbapenems) by hydrolysis of these antibiotics, and which are inhibited by β-lactamase inhibitors such as clavulanic acid.[2]

The Ambler molecular classification and the Bush-Jacoby-Medeiros functional classification are the two most commonly used classification systems for β-lactamases.[9–11] Ambler scheme divides β-lactamases into four major classes (A to D). The basis of this classification scheme rests upon protein homology (amino acid similarity) and not phenotypic characteristics. In the Ambler classification scheme, β-lactamases of classes A, C and D are serine β-lactamases. In contrast, the class B enzymes are metallo-β-lactamases. With the exception of OXA-type enzymes (which are class D enzymes), the ESBLs are of molecular class A.

The Bush-Jacoby-Medeiros classification scheme groups β-lactamases according to functional similarities (substrate and inhibitor profile). There are four main groups and multiple subgroups in this system. This classification scheme is of much more immediate relevance to the physician or microbiologist in a diagnostic laboratory because it considers β-lactamase and β-lactam substrates that are clinically relevant. In this classification, ESBLs belong to group 2be or group 2d (OXA-type), the latter sharing most of the fundamental properties of group 2be enzymes though differing in being inhibitor resistant.[10]

The 2be designation shows that these enzymes are derived from group 2b β-lactamases (for example, TEM-1, TEM-2 and SHV-1); the ‘e ’ of 2be denotes that the β-lactamases have an extended spectrum. The ESBLs derived from TEM-1, TEM-2 or SHV-1 differ from their progenitors by as few as one amino acid. This results in a profound change in the enzymatic activity of the ESBLs, so that they can now hydrolyze the third-generation cephalosporins or aztreonam (hence the extension of spectrum compared to the parent enzymes).

Inhibition by β-lactamase inhibitors such as clavulanic acid and inability to hydrolyze cephamycins differentiates the ESBLs from the AmpC-type β-lactamases (group 1), which have third-generation cephalosporins as their substrates but which are not inhibited by clavulanic acid. Selection of stably de-repressed mutants which hyperproduce the AmpC-type β-lactamases has been associated with clinical failure when third-generation cephalosporins are used to treat serious infections with organisms producing these enzymes.[12–14] In general, the fourth-generation cephalosporin, cefepime, is clinically useful against organisms producing AmpC-type β-lactamases[15] but may be less useful in treating ESBL-producing organisms.[16] Additionally, the metalloenzymes (group 3) produced by organisms such as *Stenotrophomonas maltophilia* can hydrolyze third-generation cephalosporins (and carbapenems) but are inhibited by ethylenediaminetetraacetic acid (EDTA),a heavy-metal chelator but not clavulanic acid.[17]

## EVOLUTION AND DISSEMINATION OF ESBLS

β-lactamases may be chromosomally encoded and universally present in a species or plasmid mediated. The chromosomal enzymes are believed to have evolved from PBPs with which they show same-sequence homology. This was probably a result of the selective pressure exerted by β-lactam–producing soil organisms found in the environment.[18]

The first plasmid-mediated β-lactamase in gram-negative bacteria TEM 1 was described in the early 1960s.[18] It was so designated as it was isolated from the blood culture of a named Temoniera in Greece. Being plasmid and transposon mediated, TEM-1 enzymes spread worldwide and are now found in many different species of the family Enterobacteriaceae, *Pseudomonas aeruginosa, Hemophilus influenza and Neissiria gonorrhea*. SHV-1 (for sulphydral variable type 1) is another β-lactamase commonly found in *Klebsiella* and *Escherichia coli*. Over the years, the use of newer β-lactam antibiotics has enabled selection of new variants of β-lactamases.

In the early 1980s, the third-generation, or oxy-imino, cephalosporins were introduced into clinical practice in response to the increasing prevalence and spread of the β-lactamases. Resistance to these extended-spectrum cephalosporins emerged quickly, and the first report of an SHV-2 enzyme which was capable of hydrolyzing these antibiotics was published as early as 1983 from Germany.

These enzymes were called extended-spectrum β-lactamases because of their increased spectrum of activity, especially against the oxyimino cephalosporins. There are several groups of ESBLs with similar behavior but different evolutionary histories The largest groups are the mutants of TEM and SHV β-lactamases, with over 150 members. The mutations which affect a small number of critical amino acids enlarge the enzyme ’s active site and enable it to deflect the oxyimino substitutes, which normally shield the β-lactam ring. As a result, whereas the classical TEM and SHV enzymes are unable to significantly hydrolyze the oxyimino cephalosporins, the mutants can do so, conferring resistance to their host strains.[8]

The second largest group of ESBLs is the CTX-M enzymes. Based on sequence homology, these are divided into five subgroups with around 40 members. Most of these subgroups have evolved as a result of the chromosomal β-lactamase genes escaping from Kluvera spp., an enterobacterial genus of little clinical importance. Having migrated to mobile DNA, the CTX-M β-lactamases may evolve further. Enterobacteriaceae (mostly *Escherichia coli*) producing the CTX-M enzymes have been identified, predominantly from the community, as a cause of urinary tract infections.[2–4] Various reports suggest that the CTX-M-type ESBLs may now actually be the most frequent ESBL type worldwide.[8]

The OXA-type β-lactamases (group 2d) are so named because of their oxacillin-hydrolyzing abilities. They predominantly occur in *Pseudomonas aeruginosa*[19] but have been detected in many other gram-negative bacteria.[20] The OXA-type ESBLs were originally discovered in *Pseudomonas aeruginosa* isolates from Turkey. The evolution of ESBL OXA-type β-lactamases from parent enzymes with narrower spectra has many parallels with the evolution of SHV- and TEM-type ESBLs. OXA-10 hydrolyzes (weakly) cefotaxime, ceftriaxone and aztreonam, giving most organisms reduced susceptibility to these antibiotics; but OXA-11, -14, -16, -17, -19, -15, -18, -28, -31, -32, -35 and -45 confer frank resistance to cefotaxime and sometimes ceftazidime and aztreonam.[21–26] The simultaneous production of a carbapenem-hydrolyzing metalloenzyme and an aztreonam-hydrolyzing OXA enzyme can readily lead to resistance to all β-lactam antibiotics.[26]

A variety of other β-lactamases (PER, VEB, GES.BES.TLA, SFO, IBC groups) which are plasmid-mediated or integron-associated class A enzymes have been discovered.[27–36] They are not simple point-mutant derivatives of any known β-lactamases and have been found in a wide range of geographic locations. Novel chromosomally encoded ESBLs have also been described.[37]

## METHODS FOR ESBL DETECTION

ESBL testing involves two important steps. The first is a screening test with an indicator cephalosporin which looks for resistance or diminished susceptibility, thus identifying isolates likely to be harboring ESBLs. The second one tests for synergy between an oxyimino cephalosporin and clavulanate, distinguishing isolates with ESBLs from those that are resistant for other reasons.

### Screening for ESBL producers

#### Disk-Diffusion methods

The Clinical and Laboratory Standards Institute (CLSI) has proposed disk-diffusion methods for screening for ESBL production by *Klebsiellae pneumoniae, K. oxytoca, Escherichia coli and Proteus mirabilis*. Laboratories using disk-diffusion methods for antibiotic susceptibility testing can screen for ESBL production by noting specific zone diameters which indicate a high level of suspicion for ESBL production. Cefpodoxime, ceftazidime, aztreonam, cefotaxime or ceftriaxone disks are used. Since the affinity of ESBLs for different substrates is variable, the use of more than one of these agents for screening improves the sensitivity of detection.[38] However, it is adequate to use cefotaxime, which is consistently susceptible to CTX-M; and ceftazidime, which is a consistently good substrate for TEM and SHV variants. If only one drug can be used, then the single best indicator has been found to be cefpodoxime.[8,39,40] However, it has been seen that susceptibility testing with cefpodoxime can lead to a high number of false-positive results which can be due to mechanisms other than ESBL production.[8]

If isolates show resistance or diminished susceptibility to any of these five agents, it indicates suspicion for ESBL production, and phenotypic confirmatory tests should be used to ascertain the diagnosis.

#### Screening by dilution antimicrobial susceptibility yests

The CLSI has proposed dilution methods for screening for ESBL production by *Klebsiellae pneumoniae* and *K oxytoca, Escherichia coli and Proteus mirabilis*. Ceftazidime, aztreonam, cefotaxime or ceftriaxone can be used at a screening concentration of 1 μg/mL or cefpodoxime at a concentration of 1 μg/mL for *Proteus mirabilis*; or 4 μg/mL, for the others. Growth at or above this screening antibiotic concentration is suspicious of ESBL production and is an indication for the organism to be tested by a phenotypic confirmatory test.[38]

## PHENOTYPIC CONFIRMATORY TESTS FOR ESBL PRODUCTION

### Cephalosporin/clavulanate combination disks

The CLSI advocates use of cefotaxime (30 μg) or ceftazidime (30 μg) disks with or without clavulanate (10 μg) for phenotypic confirmation of the presence of ESBLs in *Klebsiellae* and *Escherichia coli, P. mirabilis and Salmonella species*. The CLSI recommends that the disk tests be performed with confluent growth on Mueller-Hinton agar. A difference of ≥5 mm between the zone diameters of either of the cephalosporin disks and their respective cephalosporin/ clavulanate disks is taken to be phenotypic confirmation of ESBL production.[38]

For *Enterobacter spp. C freundii, Morganella, Providentia and Serratia spp*., it is better to use cefepime or cefpirome in the confirmatory tests as they are less prone to attack by the chromosomal AmpC beta lactamases, which may be induced by clavulanate in these species.[8]

### Broth microdilution

Phenotypic confirmatory testing can also be performed by broth microdilution assays using ceftazidime (0.25-128 μg/mL), ceftazidime plus clavulanic acid (0.25/4 - 128/4 μg/mL), cefotaxime (0.25-64 μg/mL), or cefotaxime plus clavulanic acid (0.25/4 - 64/4 μg/mL).[41] Broth microdilution is performed using standard methods. Phenotypic confirmation is considered as ≥3 twofold serial-dilution decreases in minimum inhibitory concentration (MIC) of either cephalosporin in the presence of clavulanic acid compared to its MIC when tested alone.

Steward and colleagues[39] suggested using cefoxitin susceptibility in isolates with positive screening tests but negative confirmatory tests as a means of deducing the mechanism of resistance. ESBL-producing isolates appear susceptible, while those with plasmid AmpC enzymes are resistant. However, resistance to cefoxitin seems to be increasing in ESBL-producing isolates due to efflux or permeability changes or coexistence of ESBLs with AmpC enzymes. The usefulness of this screen test may thus be diminishing.

### Quality control when performing screening and phenotypic confirmatory tests

Quality control recommendations are that simultaneous testing with a non–ESBL-producing organism (Escherichia coli ATCC 25922) and an ESBL-producing organism (*Klebsiella pneumoniae* ATCC 700603) also be performed.[38]

### Implications of positive phenotypic confirmatory tests

According to CLSI guidelines, isolates which have a positive phenotypic confirmatory test should be reported as resistant to all cephalosporins (except the cephamycins, cefoxitin and cefotetan) and aztreonam, regardless of the MIC of that particular cephalosporin. Penicillins (for example, piperacillin or ticarcillin) are reported as resistant regardless of MIC, but β-lactam/ β-lactamase inhibitor combinations (for example, ticarcillin-clavulanate or piperacillin-tazobactam) are reported as susceptible if MICs or zone diameters are within the appropriate range.

## OTHER METHODS AVAILABLE FOR ESBL DETECTION

Several other tests have been developed to confirm the presence of ESBLs.

### Double-disk synergy test

In this, test disks of third-generation cephalosporins and augmentin are kept 30 mm apart, center to center, on inoculated Mueller-Hinton agar (MHA).[40] A clear extension of the edge of the inhibition zone of cephalosporin towards augmentin disk is interpreted as positive for ESBL production. Evaluations of the double-disk diffusion test have revealed sensitivities of the method ranging from 79% to 97% and specificities ranging from 94% to 100%.[42–46] While the double-disk diffusion test is technically simple, the interpretation of the test is quite subjective. Sensitivity may be reduced when ESBL activity is very low, leading to wide zones of inhibition around the cephalosporin and aztreonam disks, especially for *Proteus mirabilis*.[47] False-negative results have been observed with isolates harboring SHV-2,[42–45] SHV-3[43] or TEM-12.[46] In isolates which are suspicious for harboring ESBLs but are negative using the standard distance of 30 mm between disks, the test should be repeated using closer (for example, 20 mm) or more distant (for example, 40 mm) spacing.[43–45]

A falsely positive test occurs for organisms such as *Stenotrophomonas maltophilia* because aztreonam is not a substrate for the metalloenzymes, and clavulanic acid inhibits other β-lactamases produced by this organism.[48]

### Three-dimensional test

The three-dimensional test gives phenotypic evidence of ESBL-induced inactivation of extended-spectrum cephalosporins or aztreonam without relying on demonstration of inactivation of the β-lactamases by a β-lactamase inhibitor.[45] In this test, the surface of the susceptibility plate is inoculated by standard methods for disk-diffusion testing, but additionally a circular slit is cut in the agar concentric with the margin of the plate. A heavy inoculum of the test organism (10^9^ to 10^10^ CFU of cells) is pipetted into the slit. β-lactam–impregnated disks are then placed on the surface of the agar 3 mm outside of the inoculated circular slit. β-lactamase–induced inactivation of each test antibiotic is detected by inspection of the margin of the zone of inhibition in the vicinity of its intersection with the circular three-dimensional inoculation. The presence of β-lactamase–induced drug inactivation is visualized as a distortion or discontinuity in the usually circular inhibition zone or as the production of discrete colonies in the vicinity of the inoculated slit.

### Inhibitor-potentiated disk-diffusion test

Antibiotic disks containing ceftazidime (30 μg), cefotaxime (30 μg), ceftriaxone (30 μg) and aztreonam (30 μg) are placed on the clavulanate-containing agar plates and regular clavulanate-free Mueller-Hinton agar plates.[43] A difference in β-lactam zone width of ≥10 mm in the two media was considered positive for ESBL production. A major drawback of the method is the need to freshly prepare clavulanate-containing plates. The potency of clavulanic acid begins to decrease after 72 hours.

### Cephalosporin/clavulanate combination disks on iso-sensitest agar

The British Society for Antimicrobial Chemotherapy has recommended the disk-diffusion method for phenotypic confirmation of ESBL presence using ceftazidime-clavulanate and cefotaxime-clavulanate combination disks, with semiconfluent growth on Iso-Sensitest agar (rather than confluent growth on Mueller-Hinton agar). A ratio of cephalosporin/clavulanate zone size to cephalosporin zone size of 1.5 or greater was taken to signify the presence of ESBL activity. Using this method, the sensitivity of the test for detecting ESBLs was 93% using both ceftazidime and cefotaxime. The test did not detect ESBL production by strains producing SHV-6.[49]

### Disk approximation test

Cefoxitin (inducer) disk is placed at a distance of 2.5 cm from cephalosporin disk.[47] Production of inducible β-lactamase is indicated by flattening of the zone of inhibition of the cephalosporin disk towards inducer disk by >1 mm.

## COMMERCIALLY AVAILABLE METHODS FOR ESBL DETECTION

### Vitek ESBL test

A specific card which includes tests for ESBL production has now been FDA approved. The Vitek ESBL test (bioMerieux Vitek, Hazelton, Missouri) utilizes cefotaxime and ceftazidime, alone (at 0.5 μg/mL) and in combination with clavulanic acid (4 μg/mL). Inoculation of the cards is identical to that performed for regular Vitek cards. Analysis of all wells is performed automatically once the growth control well has reached a set threshold (4-15 hours of incubation). A predetermined reduction in the growth of the cefotaxime or ceftazidime wells containing clavulanic acid, compared with the level of growth in the well with the cephalosporin alone, indicates presence of ESBL. Sensitivity and specificity of the method exceed 90%.[50]

### E Test

The E test ESBL strip (AB Biodisk, Solna, Sweden) carries two gradients: on the one end, ceftazidime; and on the opposite end, ceftazidime plus clavulanic acid.[46] MIC is interpreted as the point of intersection of the inhibition ellipse with the E test strip edge. A ratio of ceftazidime MIC to ceftazidime-clavulanic acid MIC equal to or greater than 8 indicates the presence of ESBL. The reported sensitivity of the method as a phenotypic confirmatory test for ESBLs is 87% to 100%,[43,46,51] and the specificity is 95% to 100%. The availability of cefotaxime strips, as well as ceftazidime strips, improves the ability to detect ESBL types, which preferentially hydrolyze cefotaxime, such as CTX-M–type enzymes.[2]

### MicroScan panels

MicroScan panels (Dade Behring MicroScan, Sacramento, CA.) comprise dehydrated panels for microdilution antibiotic susceptibility. Those used for ESBL detection which contain combinations of ceftazidime or cefotaxime plus β-lactamase inhibitors have received Food and Drug Administration approval; and in studies of large numbers of ESBL-producing isolates, they have appeared to be highly reliable.[52–54]

#### Becton Dickinson (BD) Phoenix Automated Microbiology System

Becton Dickinson Biosciences (Sparks, Md) have introduced a short-incubation system for bacterial identification and susceptibility testing, known as BD Phoenix.[55–57] The Phoenix ESBL test uses growth response to cefpodoxime, ceftazidime, ceftriaxone and cefotaxime, with or without clavulanic acid, to detect the production of ESBLs. The test algorithm has been delineated by Sanguinetti *et al*.[56] Results are usually available within 6 hours. The BD Phoenix ESBL detection method detected ESBL production in greater than 90% of strains genotypically confirmed to produce ESBLs. The method correctly detected ESBL production by *Enterobacter, Proteus* and *Citrobacter* spp., in addition to Klebsiellae and *Escherichia coli*.[56]

## PROBLEMS IN DETECTION

Identifying ESBL-producing organisms is a major challenge for the clinical microbiology laboratory. Multiple factors contribute to this, including production of multiple different β-lactamase types by a single bacterial isolate and the production of ESBLs by organisms that constitutively produce the AmpC β-lactamases, varying substrate affinities and the inoculum effect.

The phenotypic confirmatory tests are highly sensitive and specific compared to genotypic confirmatory tests. However, there are a number of instances whereby the phenotypic confirmatory tests may be falsely positive or negative.

*Klebsiella pneumoniae* or *Escherichia coli* isolates which lack ESBLs but which hyperproduce SHV-1 may give false-positive confirmatory test results. Such isolates can have ceftazidime MICs as high as 32 μg/mL.[58,60]

There are now numerous reports in which *Klebsiella pneumoniae* isolates have been found to harbor plasmid-mediated AmpC-type β-lactamases. Some of these organisms have been found to harbor both AmpC-type β-lactamases and ESBLs.[61] The coexistence of both enzyme types in the same strain not only results in elevated cephalosporin MICs but may also give false-negative test results for the detection of ESBLs. The likely explanation is that AmpC-type β-lactamases resist inhibition by clavulanate and hence obscure the synergistic effect of clavulanate and cephalosporins against ESBLs.

For ESBL-producing bacteria, there is a dramatic rise of MIC for extended-spectrum cephalosporins as the inoculum is increased beyond that used in routine susceptibility tests. Same isolates test susceptible at the standard inoculum and resistant at a higher inoculum. Therefore, false-negative results can occur with both screening and confirmatory tests when lower inocula are used.[41,62]

Some ESBL isolates may appear susceptible to a third-generation cephalosporin in vitro, particularly if relatively high breakpoints are used. However, treatment of infections due to an ESBL-producing organism with third-generation cephalosporins may result in clinical failure even when the MIC is below the breakpoint and the ability of these enzymes to confer resistance to weak-substrate cephalosporins is clear when MIC determinations are performed with heavy inoculum. This may be due to the variable affinity of these enzymes for different substrates and inoculum effect.[63]

Many ESBL producers are resistant to combinations despite appearing sensitive *in vitro*. This could be due to hyperproduction so that the inhibitor is overwhelmed, relative impermeability of the host or co-production of inhibitor-resistant penicillanases (e.g., OXA-1).

Since ESBL production is usually plasmid mediated, it is possible for one specimen to contain both ESBL-producing and non–ESBL-producing cells of the same species. This suggests that for optimal detection, several colonies must be tested from a primary culture plate.[64]

ESBL enzymes can be induced by certain antibiotics, amino acids or body fluids. Organisms possessing genes for inducible β-lactamases show false susceptibility if tested in the uninduced state.[47]

All these factors make detection of ESBLs a complicated and complex task, and improvements in the ability of clinical laboratories to detect ESBL are needed.

Two opposing viewpoints have arisen in recognition of the poor outcome when patients with an infection due to an ESBL-producing organism are treated with a cephalosporin to which it appears susceptible *in vitro*. Some investigators believe that alteration of cephalosporin breakpoints for *Enterobacteriaceae* by organizations such as the Clinical and Laboratory Standards Institute is a more appropriate endeavor than expanding efforts to detect ESBLs, which is too complex a task for a clinical microbiology laboratory. An advantage of such a change would be that organisms such as *Enterobacter* spp., which are not currently considered in CLSI guidelines for ESBL detection, would be covered.[2]

Another viewpoint is that the inoculum effect is important for ESBL-producing organisms. *In vitro*, the MICs of cephalosporins rise as the inoculum of ESBL-producing organisms increases.[65–68] Thus in the presence of high-inoculum infections (for example, intra-abdominal abscess, some cases of pneumonia) or infections at sites in which drug penetration may be poor (for example, meningitis, endocarditis or osteomyelitis), physicians should avoid cephalosporins if an ESBL-producing organism is present. Also severity of illness could have been greater in patients infected with organisms with higher MICs.

A point favoring efforts aimed at ESBL detection is the infection control significance of detecting plasmid-mediated multi-drug resistance. There are epidemiologic implications for the detection of ESBL-producing organisms, as the significance of this resistance may not be as apparent if organisms are simply reported as intermediate or resistant to individual cephalosporins. Outbreaks of ESBL-producing organisms can be abruptly halted using appropriate infection-control interventions. Endemic transmission of ESBL producers can also be curtailed using infection-control measures and antibiotic management interventions. Detection of ESBL production in organisms from samples such as urine may be important because this represents an epidemiologic marker of colonization (and therefore the potential for transfer of such organisms to other patients).

## RISK FACTORS

Patients at high risk for developing colonization or infection with ESBL-producing organisms are often seriously ill patients with prolonged hospital stays and in whom invasive medical devices are present (urinary catheters, endotracheal tubes, central venous lines) for a prolonged duration.[2] In addition, other risk factors have been found in individual studies, including the presence of nasogastric tubes,[69] gastrostomy or jejunostomy tubes[70,71] or arterial lines;[72,73] administration of total parenteral nutrition,[73] recent surgery,[74] hemodialysis,[75] decubitus ulcers[71] and poor nutritional status.[76]

Heavy antibiotic use is also a risk factor for acquisition of an ESBL-producing organism.[73,77,78] Several studies have found a relationship between third-generation cephalosporin use and acquisition of an ESBL-producing strain.[69,70,77–86] However, perhaps the greatest risk factor for nosocomial acquisition of an ESBL-producing organism is accommodation in a ward or room with other patients with ESBL-producing organisms.[8]

Risk factors for colonization or infection with ESBL-producing organisms, especially the CTX-M producers, include history of recent hospitalization; treatment with cephalosporins, penicillins and quinolones; age 65 years or higher; dementia: and diabetes.[2] Although there is no conclusive evidence, one potential source of colonization with the ESBL producers in the community may be the use of veterinary oxyimino cephalosporins like ceftiofur in livestock.[8]

## TREATMENT OPTIONS

The factors which determine the choice of antibiotics and other management options include a) site of infection; b) severity of infection; c) presence of a prosthetic device or implant; d) metabolic parameters — liver and renal function; e) patient-related factors such as age, pregnancy, lactation.[87] The therapeutic options for ESBL-producing organisms are very limited. ESBLs confer on them the ability to be resistant to most β-lactam antibiotics except cephamycins and carbapenems. In addition, the plasmids bearing genes-encoding ESBLs frequently also carry genes encoding resistance to other antimicrobial agents, such as aminoglycosides, trimethoprim, sulphonamides, tetracyclines and chloramphenicol.[5,8]

There have also been increasing reports of plasmid-encoded decrease in susceptibility to quinolones, frequently in association with plasmid-mediated cephalosporin resistance.[88,91] There appears to be a strong association between quinolone resistance and ESBL production,[2,92–95] even in the absence of plasmid-encoded decrease in quinolone susceptibility, although the reason for this association is not well understood. Fluoroquinolones may be used for the treatment of uncomplicated urinary tract infections (UTIs) when found to be susceptible, although increasing in vitro resistance of ESBL producers to quinolones will limit the role of these antibiotics in the future. Studies have found carbapenems to be superior to quinolones for treatment of serious infections caused by ESBL-producing organisms.[96,97]

Some infections due to organisms testing resistant to ceftazidime but susceptible to cefotaxime or ceftriaxone have responded to treatment with these alternate cephalosporins. However, MICs of these agents rise dramatically as the inoculum is increased.[98]

Thus isolates giving a positive synergy test are inferred to have ESBLs, and all cephalosporins should be avoided as therapy, irrespective of susceptibility results.

Cefamycins, such as cefoxitin and cefotetan, although active in vitro, are not recommended for treating such infections, because of the relative ease with which these strains decrease the expression of outer membrane proteins, rendering them resistant.[98,99]

Although ESBL activity is inhibited by clavulanic acid, β-lactam/ β-lactamase inhibitor combinations are not considered optimal therapy for serious infections due to ESBL producers as their clinical effectiveness against serious infections due to ESBL-producing organisms is controversial.[2] The majority of ESBL-producing organisms produce more than one β-lactamase, often in different amounts. Additionally, it is well known that ESBL-producing organisms may continue to harbor parent enzymes (for example, SHV-1 or TEM-1). Hyperproduction of these non–ESBL-producing β-lactamases[100] or the combination of β-lactamase production and porin loss can also lead to a reduction in activity of β-lactamase inhibitors.

Also β-lactam/ β-lactamase inhibitor combinations are subject to rising MICs as inoculum rises.[101] As a result, infections with high organism burden (intra-abdominal collections, sepsis) may be associated with sufficient β-lactamase production to overcome the effects of the β-lactamase inhibitor. However, they may be useful for less serious infections, such as uncomplicated non-bacteremic lower urinary tract infection, because the infection is localized and the antibiotic is excreted in large amounts through the urine.[87] They have also been found to be a good option for the treatment of uncomplicated community-acquired infections due to ESBL producers, especially since they have the advantage of oral administration.[102] The advantages of using β-lactamase inhibitors is that by inhibiting ESBLs they appear to impair the emergence and spread of *Klebsiella*-carrying resistance plasmids. Furthermore, administration of inhibitors may exert in vitro pressure on ESBLs, thereby facilitating their reverse mutation to less harmful enzymes.[98]

There is also concern that misuse of carbapenems in uncomplicated cases will result in carbapenem resistance. Thus the therapeutic potions are limited to carbapenems, colistin, polymyxin, temocillin, tigecycline for serious infections. However uncomplicated infections like non-bacteremic urinary tract infections can be managed with a variety of antibiotics, depending on their susceptibility. These include oral antibiotics like trimethoprim, nitrofurantoin, fosfomycin, co-amoxiclav, mecillinam; or intravenous agents like aminoglycoside (gentamicin, amikacin) and inhibitor combinations.[87,102] Among these carbapenems are the drugs of choice for serious infections with ESBL producers. Imipenem and meropenem are preferred in nosocomial infections, while etrapenam is preferred in community-acquired infections.[103]

Although *in vitro* studies have demonstrated no synergy, additivity or antagonism in combination therapy (carbapenem + aminoglycoside), the bactericidal activity of imipenem in combination with amikacin was found to be greater than that of imipenem alone. This was due to the faster killing rates of amikacin.[87] Thus carbapenems may be combined with a second agent (amikacin) for the first few days in the treatment of life-threatening infections like septicemia, hospital-acquired pneumonia, intra-visceral abscesses.[87] Tigecycline, temocillin, colistin and polymyxin are reserved for patients resistant to all of the other antibiotics, including the carbapenems.

## PREVENTION AND CONTROL

Proper infection-control practices and barriers are essential to prevent spreading and outbreaks of ESBL-producing bacteria. The reservoir for these bacteria seems to be the gastrointestinal tract of patients.[99] Alternative reservoirs could be the oropharynx, colonized wounds and urine. The contaminated hands and stethoscopes of healthcare providers are important factors in spreading infection between patients.[99] Essential infection-control practices should include avoiding unnecessary use of invasive devices such as indwelling urinary catheters or IV lines, hand washing by hospital personnel, increased barrier precautions, and isolation of patients colonized or infected with ESBL producers.

At an institutional level, practices that can minimize the spread of such organisms include clinical and bacteriological surveillance of patients admitted to intensive care units and antibiotic cycling; as well as policies of restriction, especially on the empirical use of broad-spectrum antimicrobial agents such as the third- and fourth-generation cephalosporins and quinolones.[2,87,99]

Some authors have suggested that use of β-lactam/ β-lactamase inhibitor combinations, rather than cephalosporins, as workhorse empirical therapy for infections suspected as being due to gram-negative bacilli, may facilitate control of ESBL producers.[104–106] However, many organisms now produce multiple β-lactamases, which may reduce the effectiveness of β-lactam/ β-lactamase inhibitor combinations.[107–110]

## CONCLUSIONS

Clinically, ESBLs limit the efficacy of β-lactams. including extended-spectrum cephalosporins, and are associated with high morbidity and mortality. Moreover, the indiscriminate use of carbapenems may select resistance to these key drugs, thus sowing seeds for significant therapeutic problems to arise in the future. There is no doubt that the ESBLs are becoming increasingly complex and diverse and their detection is becoming increasingly challenging for clinical microbiology laboratories. Thus there is need for efficient infection-control practices for containment of outbreaks; and intervention strategies, e.g., antibiotic rotation, to reduce further selection and spread of these increasingly resistant pathogens.
